# Impacts of Protecting Life in Global Health Assistance policy in Nepal: a qualitative study

**DOI:** 10.1186/s12961-023-01016-3

**Published:** 2023-06-22

**Authors:** Sarmila Dhakal, Mahesh Puri, Preeti Gautam, Kusum Wagle, Samantha Luffy, Bergen Cooper

**Affiliations:** 1 Center for Research On Environment Health and Population Activities (CREHPA), Lalitpur, Nepal; 2 Fòs Feminsta, New York, USA; 3 PAI, Washington DC, USA

**Keywords:** PLGHA, Abortion, Sexual and reproductive health, Nepal

## Abstract

**Background:**

Despite the legalization of abortion in 2002 and the concerted efforts of the Ministry of Health and Population, abortion services remain inaccessible for many Nepali women. In 2017, the United States government enacted the Protecting Life in Global Health Assistance (PLGHA) policy, which prohibited international non-governmental organizations (INGOs) from receiving United States global health assistance from providing abortion services or referrals or engaging in advocacy on liberalizing abortion laws that may have had an impact on abortion services. Though this policy was revoked in January 2021, there is a need to assess its impacts in Nepal and mitigate its lingering effects, if any.

**Methods:**

We conducted in-depth interviews with 21 national-level stakeholders selected purposively on the basis of their experiences and expertise in sexual and reproductive health and rights (SRHR) in Nepal. Interviews were conducted two times: first between August and November 2020 when PLGHA was in place, and then between July and August 2021 after PLGHA was revoked. Interviews were digitally recorded, transcribed, translated and analysed thematically.

**Results:**

Most participants reported that the implementation of PLGHA created gaps in SRHR services, affecting marginalized and underserved populations in Nepal. Participants reported that this policy has compromised the work of INGOs and civil society organizations (CSOs), posing additional risk to the sustainability of SRHR program achievements made so far. Beyond funding loss, participants also mentioned that PLGHA curtailed their freedom, with limited working areas and partnerships for CSOs leading to low or no utilization of services. Most participants welcomed the revocation of PLGHA and hoped it would have positive impacts on SRHR services by permanently repealing PLGHA. Most participants believed that the revocation of PLGHA opened opportunities for new funding and could re-establish partnerships and collaboration, though immediate results had not yet been seen.

**Conclusions:**

PLGHA had negative impacts on access to and quality of SRHR services. The Nepal government and other donor agencies need to bridge the funding gap created by the policy. The revocation of the policy has created the hope of bringing positive impacts in SRHR sector; however, the implementation of revocation at the ground level and impacts made on SRHR programs in Nepal remains to be explored.

## Introduction

Nepal amended its Penal Code (Muluki Ain) in 2002 to allow abortion on certain grounds and ensure Nepalese women’s right to make decisions on their fertility choices. Abortion was highly restrictive prior to this amendment and women were imprisoned for acts of abortion [1]. Death from unsafe abortions contributed significantly to the country’s high maternal mortality ratio [2]. The Safe Motherhood and Reproductive Health Rights (SMRHR) Act, 2018, that has replaced the Penal Code, further guaranteed women’s rights to legal and safe abortion care on wider grounds. Under this act, abortion is permitted with the consent of a pregnant woman up to 12 weeks of gestational age, and up to 28 weeks in case of (a) pregnancy resulting from rape or incest; (b) if the woman is living with HIV or other similar types of incurable diseases and desires not to continue with the pregnancy; (c) if the pregnancy poses a danger to the women’s life or affects her physical or mental health; or (d) if there is a fetal abnormality [3].

Since the conditional legalization of abortion in 2002, the Nepal government has taken important steps to provide safe and legal abortion services. In addition to recognizing abortion as women’s fundamental rights in the new constitution and the SMRHR Act, 2018, the Ministry of Health and Population (MOHP) has developed/updated regulations, strategies and directives for implementing the abortion law and expanding access to safe and legal abortion services across Nepal. The expansion of training centres along with the application of task shifting approach, training outreach health service providers such as auxiliary nurse midwives, were enabled to provide medical abortion and accreditation of their health facilities. As a result, the number of certified health facilities that provide safe abortion services has steadily expanded since 2004; by 2021, approximately 4500 clinicians (1833 auxiliary nurse midwives, 743 nurses, 1853 medical doctors and 92 obstetric and gynaecologist physicians and general practice physicians) were trained, and 1516 facilities (private as well as public) were certified [4]. In 2009, medical abortion within 9 weeks of gestation was introduced in six districts as a pilot program, and this practice has gradually scaled up to cover the entire country since then [5].

National Health Training Centre (NHTC), the sole agency of the government to provide training to health providers, started conducting training for second-trimester abortion services in 2007, and by 2021, 34 public sector and private sectors hospitals have been accredited to provide second-trimester abortions in the country [4]. By 2021, Safe Abortion Services (SAS) was available in all federal-, provincial- and municipality-level hospitals, at the majority of outreach public health facilities and at selected clinics operated by NGOs as well as private hospitals/clinics. Through all of these efforts, about 90,000 women and girls obtain safe abortion services each year [4]. The severity of complications from unsafe abortions has also drastically reduced over the years [6]. Additionally, the country’s maternal mortality ratio has declined significantly from 539 deaths per 100,000 live births in 1996 [7] to 186 deaths per 100,000 live births in 2017 [8].

Despite the MOHP’s concerted efforts to expand legal and safe abortion services, these services remain inaccessible for many Nepali women, especially those who are low income, socially marginalized or geographically isolated [9]. Of the estimated 323,200 abortions carried out in Nepal in 2014, over half of them (58%) were provided illegally [10]. Lack of awareness about the legal provisions, availability, location and cost of abortion services, as well as access to transportation to approved facilities, prevent many women from accessing obtaining safe and legal abortion services in Nepal [11, 12]. Other cultural barriers, including a lack of autonomy in reproductive decision-making due to patriarchal norms about family planning and conservative religious beliefs, also limit Nepali women’s access to legal abortion services [11–13].

Although evidence indicates that mid-level providers such as nurses and midwives can provide medical abortion as safely and effectively as physicians, the government has been slow to scale-up training for such providers, a move which could greatly expand the numbers and locations of abortion providers across the country [9]. Only 38% of public facilities permitted to provide abortion services reported offering these services in 2014 [13]. Furthermore, at that time, less than half of all public facilities that are permitted to provide post-abortion care reported doing so [13]. Fees for abortion services in private facilities are not regulated and are often prohibitively expensive [11]. The 2015 government policy of providing cost-free abortion in public facilities is an important step in this direction. However, anecdotal evidence and qualitative data suggest this policy is unevenly enforced [14].

The coronavirus disease 2019 (COVID-19) pandemic has further affected the availability and quality of abortion services in Nepal [15]. For example, one study found that one third of the total accredited safe abortion service facilities were non-functional during the COVID-19 pandemic [16].

Therefore, more investment and increased efforts to expand safe abortion services in Nepal are required to advance the country’s progress in promoting health and rights, as well as to achieve the maternal health target included in the Sustainable Development Goals (SDGs).

However, Nepal has experienced additional challenges to providing legal abortion services. In 2017, the United States government enacted the Protecting Life on Global Health Assistance (PLGHA) policy, which prohibited foreign non-governmental organizations receiving United States global health assistance from providing abortion services or referrals along with engaging in advocacy related to the liberalization of abortion laws [17]. PLGHA, which is also known as the Global Gag Rule (GGR), was applicable to nearly all forms of global health assistance including funding areas for tuberculosis, malaria, maternal and child health, HIV and AIDS, water, sanitation and hygiene, and health system strengthening [18]. The policy, however, contained exceptions for abortion services, counselling, and referrals in cases of rape, incest or if the life of a pregnant woman was at risk [19]. President Biden revoked this policy via presidential memorandum on 28 January 2021 [20].

The United States Agency for International Development (USAID) is one of the longest-standing funders of development assistance programs in Nepal [21]. USAID has a strong and collaborative partnership with the Government of Nepal with the joint goals of improving the survival and quality of life of all Nepali people through equitable and well-governed health systems. USAID has developed an integrated interventional approach to improving maternal, newborn and child health, as well as nutrition, family planning, and HIV services to maximize the impact of United States assistance in the health sector for vulnerable and hard-to-reach populations in Nepal [22].

Regressive restrictions on United States global health funding such as PLGHA could undermine Nepal’s ability to sustain its progress and meet global health targets such as the SDGs by 2030. The PLGHA policy in Nepal was in effect from 2017 to 2020. In this study, we assessed the impacts of PLGHA on sexual and reproductive health and rights (SRHR) in Nepal in its fourth year of implementation. We also documented key stakeholder’s perspectives regarding the quality of information and communication they received in the first few months after it was revoked in January 2021.

## Data and methods

This research is a part of a broader study that began in 2018 to document the impact of the PLGHA on SRHR and related health services in Nepal. Since 2018, we have assessed the impact of PLGHA on the SRHR sector in Nepal each year and the findings through 2019 have been presented elsewhere [22–24]. For the purpose of this paper, we analysed interviews conducted in 2020, the final year of the implementation of PLGHA, and in 2021 immediately after the revocation of the policy by President Biden.

We conducted in-depth interviews (IDIs) with 21 national-level stakeholders. Participants were purposively selected to capture a wide range of organizations, roles experiences and expertise relevant to the implementation of PLGHA and its revocation in Nepal. Of the 21 key stakeholders, 8 were from international organizations, 4 from national organizations including 1 anti-abortion organization, 2 from government organizations, 2 parliamentarians, 2 media representatives, and 3 representatives from UN and bilateral agencies. A total of 7 of the 21 participants reported that their organizations have received United States government (USG) funding for various programs, 6 of whom received United States global health assistance.

Among those who participated in the 2021 study, eight were also interviewed in 2019/2020. Due to COVID-19-related lockdowns and safety precautions, 15 of the 21 interviews in 2019–2020 and all 18 interviews in 2021 were conducted virtually as preferred by the participants. The in-depth interview guides included topics on: the participants’ background; their knowledge, understanding and perceptions of PLGHA; the effects of PLGHA on Nepali civil society and political and public discourse; their awareness and perception of the revocation of the PLGHA policy; and the potential impacts of the revocation on their organizations SRHR and funding. The study team participated in a 3-day intensive training on study background, objectives and methods including ethics, and members participated in regular check-in meetings throughout the project.

Each interview lasted between 30 min and 90 min. The majority of the interviews were recorded, transcribed verbatim in the respondent’s language, translated into English, and then transcripts were analysed using thematic approach. For this purpose, two researchers independently reviewed each transcript to generate a preliminary codebook that was based on the interview questions. Transcripts were then repeatedly read and coded using Dedoose. Content codes were then grouped into categories corresponding with relevant themes, including: impact of PLGHA on SRH services; impact of PLGHA on partnerships; knowledge and perceptions of PLGHA policy; and perceptions of the revocation of PLGHA policy. Key quotes that exemplified major study themes and emergent themes are presented in the results.

Additionally, we tracked seven major national daily newspapers from January to December 2020 to trace news on maternal mortality and its causes. If any maternal deaths were reported in the newspapers, we followed up on the case with relevant stakeholders to gather detailed information to assess whether such deaths were related to unsafe abortion.

The study protocol was approved by Nepal Health Research Council (reg. no. 104/2018).

## Results

Participants had diverse roles with regard to national policymaking and program implementation, including health service provision, evaluation, research and advocacy related to SRHR. We identified four main themes related to PLGHA: impact of PLGHA’s implementation on sexual and reproductive health (SRH) services, impact of the policy on civil society organizations, knowledge and perception of the PLGHA policy, and perception of the revocation of the PLGHA policy.

### Impact of PLGHA on SRH services

Several participants (15 out of 21) shared that the implementation of PLGHA created gaps in SRHR service availability and utilization, which predominantly affected marginalized and underserved populations in Nepal. These participants noted that the cuts in United States government funding reduced SRHR program activities such as demand-generation activities and the supply of equipment, commodities and training for service providers.

Participants stated that family planning and safe abortion services were the two most affected SRH services. Most of the participants (19 out of 21) expressed a perception that the funding that had been cut off due to the PLGHA policy had a direct impact on the ability of organizations to deliver SRH care in Nepal, and six organizations that provide SRH services organizations had reported experiencing funding cuts. Participants voiced that organizations had to cut off all the programs and activities related to abortion to continue to receive funds from USAID and sustain their organizations. This has created gaps in service availability and utilization in health institutions run by both government and private sectors. Stakeholders reported organizations had to curtail their staff and scale down their programs or close them early, which hindered their ability to provide services.

Between January and December 2020, 34 maternal deaths were reported in the major Nepali newspapers. Of the total deaths, two were related to unsafe abortion. Investigations of these two cases indicated that there was only one public hospital providing safe abortion services since support for the provision of safe abortion services from private and non-profit organizations had either declined or stopped completely since 2017. Limited access to safe abortion services might have compelled women to use unsafe methods if they were unable to travel long distances for legal abortion services.

As one NGO participant reported:

*‘First and foremost, the policy will impact women of a marginalized and poor community; those who can afford [abortion] will have access to the service anyhow. With the support of organizations, [and] to some extent the public health facilities in the community are able to provide family planning services, with the help of (Female Community Health Volunteer), family planning services and knowledge are accessible in the community. If the service discontinues, the marginalized communities dependent on it will have a difficult situation.’*

-ID 14, NGO working on SRHR

Participants also felt that since Nepal depends on donor funding for SRHR services, specifically United States global health assistance, PLGHA would compromise the work of national non-governmental and civil society organizations and pose additional risks to the sustainability of achievements made in relation to SRHR so far. The majority of the respondents (16 out of 21) explained that Nepal’s health system, which is already strained and fragile, is facing a double threat to SRHR outcomes: the current COVID-19 pandemic layered on top of the implementation of the Trump administration’s extended GGR policy and the administration’s defunding of the United Nations Population Fund (UNFPA) due to an unsubstantiated Kemp-Kasten Amendment violation [26]. Participants noted that the restrictive PLGHA policy decreased bilateral resources in the SRHR sector in a situation where a large portion of national government resources was focused on COVID-19-related preparedness and response.

One participant described the impact of COVID-19 and the loss of United States funding for SRH programs in Nepal in this way:

*‘Due to the COVID-19 pandemic, the international fund on safe abortion for 2021 has been declined by 35%. Therefore, there is a challenge for us to implement a safe abortion program in 2021…I have heard the SRH clinics are on verge of being closed down...For us, a 35% decline in the fund [means] we may not be able to carry out our safe abortion program on the large scale. In this situation, we are not eligible to apply for any USAID funding opportunities due to the policy.’*

-ID 12, NGO working on SRHR

Few participants reported that organizations had to divert their programmatic focus from health, particularly SRHR, to other causes due to defunding and the operational challenges they were facing when PLGHA was in effect. However, two of the participants emphasized that it is more challenging for national NGOs than INGOs to divert programmatic focus to issues other than SRHR issues due to limited capacity in terms of resources and skills. A representative from a national organization that does not receive United States funding due to PLGHA described the difficulty their organization has faced in seeking new funding opportunities as follows:

*‘We are banned from new USAID opportunities because we provide safe abortion services. It is very difficult for us to explore new opportunities as well, so there has been a huge impact.’*

-ID 12, NGO working on SRHR

### Impact of PLGHA on partnerships

Several respondents reported that the PLGHA policy impacted partnerships between organizations supporting safe abortion and the organizations receiving USG funds in Nepal, since national organizations were compelled to choose to work either with USG funding recipients or with those focusing on abortion. One participant stressed that the organizations working on safe abortion had a hard time finding district-level partners to implement programs related to abortion at the community level.

Participants reported that the organizations working to expand safe abortion services in Nepal were not allowed to work with USAID-funded organizations or receive USAID funds when PLGHA was in effect from 2017 to 2021. During interviews, participants highlighted the financial dilemma that their organizations faced regarding whether to receive USAID funding and not work in abortion-related activities or choose other sources of funds to continue working on safe abortion services and losing opportunities to work with USAID. A participant from a prime implementing partner described an incident where one of their local sub-prime partners had to reject an opportunity to implement a program that included safe abortion services with support from another donor in a district that had poor maternal and child health outcomes in order to continue implementing programs with their United States funding. He said:

*‘One of our local partners in Bajura was selected for an abortion-related project but they could not accept the funds being a complaint organization. The decision was not easy, Bajura has a high prevalence of child marriage, women get pregnant at an early age, and access to abortion service would have supported a lot. The organization requested for a consideration so that they could run both of the projects, however, we could not support their request.’*

-ID 17, INGO, USG Prime Recipient

One of the most devastating impacts of PLGHA that many participants shared was the early closure of a large USG-funded program called Support for International Family Planning Organization (SIFPO-II), which was the largest family planning program funded by USAID and implemented by two organizations through their branch offices in 22 districts of Nepal. As the prime implementing partners did not comply with the PLGHA policy after it was implemented in 2017, SIFPO-II had to be terminated before the program was complete. This also meant that the organizations losing funding due to PLGHA had to halt or scale down their program activities, cut staff positions and remain compromised. A representative from an NGO organization providing safe abortion services, and who therefore could not receive United States funding when PLGHA was in effect stated:

*‘SIFPO-II was a family planning and [health] system strengthening project of the government. Due to the GGR, now, we don’t have [any] major family planning projects and we also have a funding crisis. We are not able to scale up the family planning program. For example, we have been organizing a vasectomy camp from our core funding and it is limited. As we have limited resources, there has been an impact on the large-scale programs aimed at increasing the couple years of protection. Likewise, we have been trying to explore new family planning projects but we are backed away from USAID funds…we are banned from new USAID opportunities because we provide safe abortion services.’*

-ID 12, NGO working on SRHR

Apart from USAID funding, participants also highlighted that the PLGHA policy dismantled opportunities of organizations, particularly organizations that were compliant and non-compliant with PLGHA, to work together and support the Nepali government in the emergency response to COVID-19. For instance, a government representative reported that it was challenging to collaborate with a few international non-governmental organizations (INGOs) due to PLGHA restrictions, which influenced the government’s ability to implement interim guidelines developed in May 2022 to support reproductive, maternal, neonatal, child and adolescent health services at all levels of health facilities during the COVID-19 pandemic [25].

A representative from a UN agency reported the impacts of funding cuts for SRHR services for national NGOs compared with INGOs in this way:

*‘If funding is cut for the health sector and for reproductive health and family planning services, then I think the situation of the NGO becomes more limited, their coverage is limited which directly impacts their work. They might be diverting their focus from SRH services to other health sectors. INGOs seem to be more resilient to such issues but the local NGOs are more vulnerable.’*

-ID 13, UN Agency

Participants from organizations receiving USG funding also expressed reluctance in joining any meetings, trainings, or workshops, which would include abortion-related discussions when PLGHA was in effect. One participant shared that the avoidance of participating in such meetings and fora were mainly to build and maintain a good relationship with USAID. They said:

*‘…we refrain ourselves from abortion-related events. We can join the meetings in reproductive health like we join the reproductive health sub-cluster meetings but if an organization that works in safe abortion requests us to be a part of the abortion-related discussion and give our opinion in such instances; we will not be able to join them.’*

-ID 17, INGO, USG Prime Recipient

Another NGO representative described organizations’ hesitation to engage in meetings to preserve their relationship with USAID in this way:

*‘While I was working in XXX [name of the organization], our invitation for dissemination and meetings were denied by many of the organizations [that were compliant with PLGHA]. The ignorance is not due to their current position, but the avoidance is to strengthen the future relationship with USAID, avoiding such meeting makes them eligible for future collaboration with USAID.’*

-ID 06, INGO working on SRHR

A representative from an NGO that does not receive USG funding also reported observing a difference in partners’ ability to engage in SRHR advocacy if they were compliant with PLGHA when it was in effect:

*‘We can see differences among the organizations, for example, organizations who worked on SRHR rights and advocacy initially now have accepted USAID funds and they are hesitant to speak on SRHR rights.’*

-ID 12, NGO working on SRHR

One of the participants further noted that when people representing two different institutions (one working for safe abortion and the other working with USAID funds) attend national-level events, they were self-segregated into groups when PLGHA was in effect.

*‘During meetings/workshops, we have heard people saying, “This table belongs to abortion.” Those who have been working with USAID for a long time hesitate to share a table with us during workshops and meetings. Similarly, the USAID-funded organization at the local level denied our partnership few years back. Even though they were interested to work with us, the GGR policy did not allow them to have a partnership. There were 3-4 organizations who had denied working with us due to the policy.’*

-ID 03, INGO working on SRHR

### Knowledge and perception on PLGHA policy

Regardless of whether the PLGHA was in effect or not, knowledge about the policy among the participants was poor. Only two of the respondents, one representing a PLGHA-compliant organization and the other from a non-compliant organization, correctly explained the PLGHA policy in detail during their interview. The majority of the participants described it as a policy that does not support abortion and prevented USAID from supporting organizations working on safe abortions. Two of the participants (a representative from a bilateral organization and a parliamentarian) did not know about the policy at all when they were interviewed. One INGO staff person described people’s knowledge and understanding of the policy in this way:

*‘We work with media partners and as per my observation, media people have very little knowledge of GGR. Only a few who cover health-related news are known to [aware of] the policy here in the capital city. If not, I do not think the media can assess the impact of the policy and cover the issue. And also there is very little news coverage on the GGR policy and when covered, it is interpreted in the wrong way.’*

-ID 03, INGO working on SRHR

Three stakeholders perceived that their partner organizations were confused about PLGHA and misinterpreted the policy when it was in effect. They shared instances in which their partner organizations were restricting themselves more than the clause of the policy required, a phenomenon known as the chilling effect [18, 26]. Participants were also confused about whether referrals for safe abortion were allowed under the policy.

*‘There is a lot of confusion. The policy restricts abortion services and advocacy but not referrals; here the compliant organizations are also restricting the referrals.’*

-ID 06, INGO working on SRHR

Most of the participants (18 of 21) did not support the PLGHA policy. Almost all participants perceived PLGHA as going against the constitution and national law of Nepal, which supports the provision of safe and legal abortion services. Almost all participants explained that PLGHA was not appropriate in the context of Nepal as it could halt the progress achieved in women’s health, including maternal and child health. These participants also described the moral dilemma they were experiencing when PLGHA was in effect regarding the ignoring of safe abortion services although they were prioritized by national policy and strategies. A participant from an organization receiving USG funds stated:

*‘The policy sounds conflicting and contradicts to what is already allowed in our country…civil service organizations are being cramped by the foreign policy which is against the legal provision of [safe abortion] in Nepal and these instances will definitely create an impact.’*

-ID 01, NGO working on women’s health, USG Recipient

Respondents also expressed that the prime implementing partners receiving USG funding had an additional burden, as they had to increase their efforts to train staff, ensure they were aware of PLGHA and monitor their compliance with the policy in the field.

*‘We provided refresher training on the PLGHA course to our sub grantee. However, it demands additional effort; there was a need for additional resources. We had to develop training material including the PLGHA clauses. The training manual had to prepared according to the cadre, for example, the key staff would need to know the policy in detail, whereas the front line had to be well aware of certain clauses.’*

-ID 17, INGO, USG Prime Recipient

### Perception of the revocation of PLGHA

The majority of the participants in the 2021 interviews (16 of 18) welcomed the revocation of the PLGHA policy in January 2021 and considered it a milestone in the advancement of women’s and girls’ SRHR. Though it was too early to measure the impact of the revocation when these interviews took place in August 2021, most of the participants expressed that the ‘rays of hope’ coming from the revocation can signal a renewed commitment to women’s rights and health. However, respondents also expressed the desire for a permanent solution to back-and-forth implementation of the GGR as it is the policy being regularly implemented by Republican presidents and revoked by Democratic presidents in the United States. The participants highlighted that access to safe abortion should not be a political topic that is affected by the people in power. Participants expressed their reaction to the revocation of PLGHA and their support for a permanent end to the GGR in this way:

*‘This is obviously good for us. This revocation has provided a kind of relief to organizations working on SRHR like us. Because there has been a lot of indirect impact of GGR policy since this was implemented and now all these are waved out. With this revocation, we have moral support now to work on the SRHR sector. Of course, the financial support will also increase. But, most importantly we will have both the financial support and also moral support to work on SRHR issues of women in Nepal…When the Democrats win, they revoke the policy and when Republicans wins, they again implement the policy. This should be removed forever and should no more be a political agenda.’*

-ID 06, INGO working on SRHR

*‘I assume that support in terms of funds will be increased. Along with that, there might be new family planning programs in place to support us. After revocation, if any family planning projects announced, it will be a great support to us, particularly in this situation of COVID-19.’*

-ID 20, Senior Government Officer

Alternatively, one participant from a faith-based organization in Nepal strongly opposed the policy’s revocation because their organization does not support abortion.

Most worryingly, most (10 out of 18) participants when PLGHA was revoked reported receiving no formal communication about the revocation of the policy from the United States funding agency or other organizations, such as their central office or headquarters. One participant had not even heard about the revocation before the interview while eight of them had heard about it indirectly through national and international media.

*‘I did not receive any official information from the organization I am working with. As far as I remember, I think I have received a generalized kind of email mentioning about the revoke of this policy. I did not receive any guidance or email on detail about what has been changed. In addition, mostly, I learnt about it from the news.’*

-ID 02, INGO, USG Prime Recipient

Most of the participants have not observed or did not know any organizations who are continuing to implement the policy since it was revoked. However, two participants shared that there might be some possibilities to the continuation of the implementation.

*‘US grantees may not be fully aware of the revocation and the ongoing programs are still subjected to restrictions imposed earlier.’*

-ID 13, UN Agency

Those who had received direct communication related to the revocation did so through email and discussions in meetings. One participant described these communications as follows:

*‘We have received communication from our head office that those restrictions have now been revoked and we should start looking for opportunities to bid for any call that may be published.’*

-ID 08, INGO working on SRHR

## Discussion and conclusions

This study examined the impacts of the PLGHA in its fourth year of implementation in Nepal, as well as documented key stakeholder’s perspectives in the first few months after the policy’s revocation in January 2021.

Participants described the widespread impacts of the interruption in funding due to PLGHA when it was in effect on the delivery of SRH services in Nepal through 2020. Participants reported that the policy was restricting women’s rights and their ability to access SRH services such as family planning and safe abortion services and disproportionately affected the rural, poor, illiterate and most marginalized communities of Nepal. These findings are consistent with other research; the Ministry of Health and Population reported that the modern contraceptive prevalence rate in Nepal decreased from 44% in 2016/2017 to 37% in 2019/2020 to 39% in 2020/2021 and that the number of adolescents (less than 20 years old) receiving safe abortion services decreased in 2019/2020 compared with the previous year [4]. Though it is not possible to determine a causal relationship between this decline in family planning and abortion services with the implementation of PLGHA, there are indications that the closure of clinics run by the organizations funded by USAID likely contributed to this decline [27].

These findings also indicated that PLGHA made it difficult for organizations to work in partnerships. This has not only undermined SRHR advocacy efforts and the provision of SRH services, but also contributed to a duplication of efforts and the waste of limited resources [28–30]. For instance, many local organizations that were complaint with PLGHA did not refer women to non-certifying organizations or facilities to receive SRH services in Nepal and Kenya when the policy was in effect [27, 31]. In Nepal, family planning programs have been integrated into maternal, neonatal and child health programs since the third Five-Year Plan (1965–1970) [32, 33]. Several other studies have demonstrated positive impact by integrating family planning into other health services such as immunization [33], nutrition [34] and HIV and AIDS [35]. Therefore, the disruption of one aspect of SRH programs (for example family planning services) by the PLGHA policy could halt the progress on other aspects including maternal and child health, nutrition and HIV and AIDS.

Similar to the findings of our study, misinterpretation and over-implementation of the policy was found not just during Trump’s administration, but also in the version of the GGR that was instituted by previous Republican president as well. Several studies reported a lack of clear understanding of the policy since it was first implemented in 1984 [18]. Similar to our study, people in Brazil were not sure whether they would be invited for workshops and trainings supported by USG funding assistance if they advocated for abortion laws [36]. The confusion and fear was more common among the sub-prime organizations as compared to prime non-implementing organizations [37]. A scoping review found that confusion and misinformation could be generated when there are multiple interacting levels of the health system [18]. For instance, imposing PLGHA in a country where abortion is permitted upon request can lead to confusion and fear while the service providers negotiate between local law and PLGHA compliance [18]. The same scenario might have induced the chilling effect, including miscommunication, misinterpretation and confusion, among the participants in this study, as Nepal has legalized and is providing safe abortion services since 2002 [3].

This study contributes new insights to the issue of the implementation and revocation of the PLGHA policy in Nepal. Rich and contextual information was gathered from participants having diverse work experience in SRH in Nepal. However, because of our limitation in terms of cost and time, we could not include all the stakeholders working in the sector of SRH in Nepal and thus had to limit the number of participants. Future studies could explore whether the findings of this study are similar to those reported by stakeholders working in health sectors including malaria, tuberculosis, MCH, HIV and AIDS, Water, Sanitation, and Hygiene (WASH), and health system strengthening to understand the impact of PLGHA on those sectors as well in Nepal. In addition, future research could incorporate quantitative methods to document the long-term impacts of PLGHA and identify what can be done to mitigate and prevent such impacts if the policy is reinstated in the future.

Now that President Biden has revoked the PLGHA policy, the results of the study could be useful to advocates and policymakers to understand the impact of the PLGHA policy in Nepal, including the impact of unclear and insufficient communications about the revocation. Importantly, this study documented the perspectives of the stakeholders immediately after the revocation of the policy, so it limits our examination of the lingering impact of the policy after its revocation. Further studies could examine whether there were continued impacts of PLGHA in a lower-middle-income country such as Nepal after its revocation.

Civil society organizations are the key players in the development sector, providing opportunities to bring communities together for collective action, mobilizing society to articulate demands and voice concerns at local, national, regional and international levels to advance health and rights. Therefore, they should advocate and support global efforts to push the United Sates Congress to permanently repeal the PLGHA and other abortion-related restrictions on United States foreign assistance such as the Helms Amendment through legislative action, and remain informed and communicate with partners and staff about the revocation of PLGHA by United States President Biden and its implication on USG funding.

The Government of Nepal needs to identify the scale and gravity of the impacts of PLGHA and work to address the SRH service availability and accessibility gaps that were created by the policy in the health sector. The government could create a platform inviting organizations working in SRHR and funding agencies to discuss the impact of PLGHA and identify mitigating measures, as now the policy has been revoked by President Biden. The government should develop proactive strategies to protect and support CSOs working in Nepal and facilitate bilateral and philanthropic organizations to support and fund the organizations working in SRHR in Nepal.

## Data Availability

De-identified data used and analysed in this study will be available from the corresponding author on reasonable request.
